# A Reasonable Evaluation of *Chuanxiong Rhizoma* Processing with Wine through Comparative Pharmacokinetic Study of Bioactive Components: Dominant Effect on Middle Cerebral Artery Occlusion Model Rats

**DOI:** 10.1155/2022/8252038

**Published:** 2022-03-14

**Authors:** Ke Pei, Lilong Cao, Gang Cao, Hao Cai, Yan Ning, Tingting Zhao, Lin Sun, Haixin Liu, Shuosheng Zhang

**Affiliations:** ^1^Shanxi Engineering Laboratory of Modern Chinese Medicine, School of Chinese Medicine and Food Engineering, Shanxi University of Chinese Medicine, Jinzhong, China; ^2^Research Center of TCM Processing Technology, Zhejiang Chinese Medical University, Hangzhou, China; ^3^School of Pharmacy, Nanjing University of Chinese Medicine, Nanjing, China

## Abstract

According to the ancient documents and Chinese herbal medicine processing experience, *Chuanxiong Rhizoma* was usually processed with yellow rice wine to improve efficacy. However, the relevant mechanisms are still unclear so far. In this study, a validated ultrahigh-performance liquid chromatography tandem mass spectrometry method was used to compare the pharmacokinetics of four representative components in middle cerebral artery occlusion rats after oral administration of raw and wine-processed *Chuanxiong Rhizoma*. The neurobehavioral scores and 2,3,5-triphenyltetrazolium chloride staining were employed to evaluate the model. Biological samples were prepared by protein precipitation with methanol. All analytes were separated on an ACQUITY BEH C_18_ column through gradient elution using acetonitrile and 0.01% of formic acid as mobile phase, and the flow rate was 0.3 mL/min. The results showed that the maximum plasma concentrations, the area values under the concentration-time curves of senkyunolide A, and ferulic acid in wine-processed *Chuanxiong Rhizoma* were all higher than in raw *Chuanxiong Rhizoma*, which were completely opposite to our previous studies in normal rats. Compared with normal rats, the theory that wine processing could enhance the efficacy of *Chuanxiong Rhizoma* may be better reflected in model rats.

## 1. Introduction

*Chuanxiong Rhizoma*, the dried rhizome of *Ligusticum chuanxiong* Hort., a commonly used traditional Chinese medicine (TCM), has a function of promoting blood circulation and removing blood stasis [1], usually used for cardiovascular and cerebrovascular system diseases [2–4], headache [5], and menstrual symptoms [6]. Modern studies have demonstrated that it also has other pharmacological activities such as antioxidant [7, 8], neuroprotection [9], antifibrosis [10], antinociception [11], anti-inflammation [12–14], and anticancer activities [15].

Wine processing is a common pretreatment procedure of *Chuanxiong Rhizoma*, which appeared as early as on Song dynasty, widely applied in Ming and Qing, and is still in use today. It is generally assumed that wine-processed *Chuanxiong Rhizoma* (WCR) have a better effect in activating blood circulation, removing blood stasis, analgesia, and so on compared to raw *Chuanxiong Rhizoma* (RCR) [16, 17]. However, the relevant mechanism remains unclear. The main components of *Chuanxiong Rhizoma* contain phthalides, alkaloids, organic acids, esters, and polysaccharides. Butylidenephthalide, ligustilide, and senkyunolide A are all phthalide components that primarily existed in volatile oils [18, 19]. Butylidenephthalide has been approved by the China Food and Drug Administration for the treatment of acute ischemic stroke. Ligustilide has effect of vasodilatation and neuroprotection. Senkyunolide A can inhibit the formation of thrombi, increase cerebral blood flow, and reduce cerebral vascular resistance [20]. Ferulic acid is a phenolic acid with the effect of ameliorating nerve injury induced by cerebral ischemia in rats [21], inhibiting platelet [22] and anti-inflammation [23], which is used as a marker to control the quality of *Chuanxiong Rhizoma* in the Chinese Pharmacopoeia (Edition 2020) [1]. Our previous study found that, by comparing to RCR, the contents of butylidenephthalide, ligustilide, senkyunolide A, and ferulic acid were increased in WCR; however, the *C*_max_ and AUC values in normal rats after oral administration WCR were all decreased [24]. Facing such contradictory results, we thought that the pharmacokinetic processes of active ingredients in model animals may be different. Therefore, the four compounds were selected as the target analytes in our experiments. *Chuanxiong Rhizoma* and its preparations are often used in the clinical treatment of stroke; thus, middle cerebral artery occlusion (MCAO) model rats were established to investigate the plasma pharmacokinetics in this study.

Q Exactive Focus Orbitrap mass system equipped with high selective performance of quadrupole and high resolving power of Orbitrap has advantages of superior accuracy (10^−7^–10^−6^), high resolution (up to 10^6^), and excellent sensitivity [25]. In this study, a rapid, specific, and sensitive ultrahigh performance liquid chromatography tandem mass spectrometry (UHPLC-MS/MS) method has been developed for the simultaneous determination of four analytes in MCAO model rat plasma. The obtained results would be helpful for understanding the wine-processing mechanism of *Chuanxiong Rhizoma* and guiding the clinical treatment in cerebral ischemia stroke.

## 2. Materials and Methods

### 2.1. Chemicals and Reagents

The reference of the analytes (butylidenephthalide, ligustilide, senkyunolide A, and ferulic acid) was purchased from Chengdu Herbpurify Co., Ltd (Chengdu, China). Internal standard (IS) calycosin-7-*O*-*β*-D-glucoside was provided by Shanghai Standard Technology Co., Ltd (Shanghai, China). The purity of each reference standard was above 98%. Acetonitrile and methanol (LC-MS grade) were obtained from Thermo Fisher Technology Co., Ltd (Fair Lawn, NJ, USA). HPLC grade formic acid was purchased from Tianjin Guangfu Fine Chemical Research Institute (Tianjin, China). The yellow rice wine was supplied from Shaoxing Wuyue Brewing Co., Ltd (Shaoxing, China). Deionized water was purified with a Milli-Q system (Millipore, Bedford, MA, USA).

### 2.2. Preparation of WCR, Oral Liquid Medicine of RCR, and WCR

*Chuanxiong Rhizoma* was indigenously cultivated in Sichuan province and authenticated by Prof. Shuosheng Zhang. The preparation methods of WCR, oral liquid medicine of RCR, and WCR have been established in our previously published research article [24], and generally, 10–20 kg of wine is used for every 100 kg of medicinal materials.

### 2.3. Animals

Male Sprague Dawley rats, weighing 220–250 g, were purchased from the Laboratory Animal Center of Shanxi Medical University (License No. SCXK (Jin) 2019–0004; Taiyuan, China). The rats were kept in an air-conditioned laboratory animal center under a 12/12 h light/dark cycle for 7 days, with the temperature maintained at 22–26°C and the relative humidity at 60 ± 10%. All rats were fasted with free access to water overnight before the experiment. All procedures were performed according to the Regulations of Experimental Animal Administration published by the State Committee of Science and Technology of People's Republic of China. All attempts were made to reduce the use of animals and their suffering.

### 2.4. Establishment and Evaluation of MCAO Model Rats

After one-week acclimatization, 24 rats were randomly divided into two groups: sham operation group (*n* = 6) and MCAO model group (*n* = 18). Transient focal cerebral ischemia was induced by intraluminal middle cerebral artery occlusion as previously described [26, 27]. Rats were anesthetized by intraperitoneal injection of 1% pentobarbital sodium at a dose of 30 mg/kg and placed on the heating cushion to maintain body temperature at 37°C. A neck midline incision was made to expose and separate the right common carotid artery (CCA), external carotid artery (ECA), and internal carotid artery (ICA). The right external carotid artery was ligated. A nylon monofilament (diameter 0.25–0.28 mm) coated with silicone tip was inserted from the ECA into the lumen of the ICA, nearly 17.5–18.5 mm from the bifurcation, to block the middle cerebral artery (MCA). Following a 2-h induction of ischemia, the filament was slowly withdrawn to allow reperfusion and the wound was surgically sutured. Rats in the sham group only underwent the same operation described above except for the insertion of nylon monofilament. We applied all laboratory procedures to minimize rat sufferings such as heating pad, sterilization, and fluid replenishment with normal saline.

The neurological function was determined at 24 h after reperfusion according to the Zea-Longa score method to evaluate the establishment of the MCAO model [26]. The scoring criteria are as follows: 0 point, behavior is completely normal, without any symptoms of neurological deficit; 1 point, mild neurological deficit, dysfunction in stretching the left forelimb; 2 points, moderate neurological deficit, rats cannot go straight and walk forward because the body continues to turn to the side; 3 points, severe neurological deficit, rats cannot stand and fall to the left when standing; 4 points, rats cannot spontaneously walk, loss of consciousness; and 5 points, rats die. The higher the score, the more serious the neurobehavioral dysfunction. The rats with scores ranging from 1 to 4 were selected for the pharmacokinetic experiment.

After the evaluation of neurobehavioral scores, the brain tissues of 6 rats in the model group and all rats in the sham group were carefully removed, washed with cold PBS, and immediately frozen at −20°C for 20 min. The brain tissues were then dissected into 6 slices along the coronal plane from the frontal lobe (2 mm thick). The slices were incubated in 2% 2,3,5-triphenyltetrazolium chloride (TTC) at 37°C for 30 min. The infarct regions were stained white, whereas the normal tissues were stained red. The slices stained were washed three times with saline, fixed with 4% paraformaldehyde solution overnight, and then photographed. Image *J* software (National Institutes of Health, Bethesda, MD, USA) was used to measure the cerebral infarct area. The infarct volume was calculated as follows: infarcted area/total brain area × 100%. Later, the rest of the MCAO model rats were randomly divided into two groups, namely, orally administrated with RCR and WCR, respectively.

### 2.5. Instruments and Analytical Conditions

A quadrupole-Orbitrap mass spectrometric detector equipped with an electrospray ionization (ESI) interface (Thermo Fisher Scientific, San Jose, CA, USA) was used for quantitative and qualitative analyses. Chromatography separation was performed by using a Dionex Ultimate 3000 ultrahigh performance liquid chromatography system (Thermo Fisher Scientific, Sunnyvale, CA, USA) consisting of a binary pump (HPG-3400RS), an autosampler (WPS-3000), a column compartment (TCC-3000), and an online degasser.

The separation was performed on an ACQUITY BEH C_18_ column (100 mm × 2.1 mm, 1.7 *μ*m; Waters, MA, USA), which was maintained at 40°C during the analysis. The mobile phase consisted of acetonitrile (*A*) and 0.01% (*v/v*) aqueous formic acid (B). In order to compare the pharmacokinetic behaviors of these compounds in normal rats, the gradient elution procedure and mass spectrometry detection parameters were the same as our previous research [24].

### 2.6. Preparation of Standard Solutions, Calibration Samples, and Quality Control (QC) Samples

The stock solutions of butylidenephthalide, ligustilide, senkyunolide A, and ferulic acid were separately prepared in methanol at 190, 260, 240, and 260 µg/mL. The working solutions were obtained by mixing each stock solution together and serially diluting to different concentrations. Calycosin‐7‐O‐*β*‐D‐glucoside (IS) was dissolved in methanol to prepare the stock solution at 280 *μ*g/mL and then diluted to an appropriate concentration of 1,400 ng/mL. All solutions were kept at 4°C before use.

The calibration standard samples were prepared by spiking 10 *μ*L of working solution into 100 *μ*L of blank plasma to obtain final concentrations of 2.38, 4.75, 9.50, 47.50, 95.00, 475.00, and 950.00 ng/mL for butylidenephthalide; 3.25, 6.50, 13.00, 65.00, 130.00, 650.00, and 1300.00 ng/mL for ligustilide; 3.00, 6.00, 12.00, 60.00, 120.00, 600.00, and 1,200.00 ng/mL for senkyunolide A; and 6.50, 13.00, 26.00, 130.00, 260.00, 1,300.00, 2,600.00, and 26,000.00 ng/mL for ferulic acid.

The QC samples were prepared in the same way above. The low-, middle-, and high-concentration QC samples of each component were 4.75, 95.00, and 760.00 ng/mL for butylidenephthalide; 6.50, 130.00, and 1,040.00 ng/mL for ligustilide; 6.00, 120.00, and 960.00 ng/mL for senkyunolide A; and 13.00, 1,300.00, and 20,800.00 ng/mL for ferulic acid.

### 2.7. Sample Preparations

The plasma sample (100 *μ*L) was mixed with 10 *μ*L of IS (140 ng/mL) and 10 *μ*L of methanol by vortexing for 3 min. Subsequently, 300 µL of methanol was added for protein precipitation. The mixture was vortexed for 5 min and then centrifuged at 13,000 rpm for 20 min. The supernatant was centrifuged again, and 5 *μ*L of the solution was injected into the UHPLC-MS/MS system for analysis.

## 3. Method Validation

### 3.1. Specificity

The specificity of the method was validated by comparing the chromatograms of blank plasma from six rats, blank plasma spiked with analytes at LLOQ, and IS and actual plasma samples after oral administration of RCR.

### 3.2. Linearity and Lower Limit of Quantification (LLOQ)

The calibration curves were fitted by plotting the peak area ratio of each analyte to IS (*y*) versus plasma concentrations (*x*) using a weighted least-squares linear regression. The LLOQ was determined as the lowest concentration on the calibration curve, at which precision (RSD) and accuracy (RE) should be less than 20% according to guidance.

### 3.3. Precision and Accuracy

For the evaluation of intraday precision and accuracy, six replicates of QC samples were analyzed at three concentration levels on the same day. For the evaluation of interday precision and accuracy, six replicates of QC samples were analyzed at three concentration levels on three consecutive days. Precision was expressed by the relative standard deviation (RSD). Accuracy was assessed by comparing the measured concentration to the theoretical concentration and expressed as the relative error (RE). The measured concentration of each QC sample was calculated using the regression equation of calibration curves prepared on the same day. It is acceptable that the RSD value is less than 15% and that the RE value is within ±15%.

### 3.4. Extraction Recovery and Matrix Effect

The extraction recovery was evaluated by comparing the peak areas of QC samples that were spiked with analytes prior to extraction with a neat standard solution at equivalent concentration. The extraction recovery of IS was analyzed in the same way. The matrix effect was assessed by comparing the peak areas of QC samples that were spiked with analytes postextraction with a neat standard solution at equivalent concentration.

### 3.5. Stability

The stability was evaluated by analyzing QC samples at three concentration levels under the following conditions: at room temperature for 4 h before sample preparation (short-term stability), at 4°C for 12 h in the autosampler after sample preparation (postpreparative stability), after three freeze (−20°C)-thaw (room temperature) cycles (freeze-thaw stability), and at −20°C for 60 days (long-term stability). The samples were considered stable when RSD was within ±15%.

### 3.6. Pharmacokinetic Study

After the MCAO model was successfully established, rats in the RCR group and WCR group were orally administered with liquid medicine of RCR and WCR, respectively, at 24.01 g/kg (equivalent to 0.19 mg/kg of butylidenephthalide, 0.07 mg/kg of ligustilide, 2.79 mg/kg of senkyunolide A, and 0.33 mg/kg of ferulic acid for RCR, while 0.23 mg/kg of butylidenephthalide, 0.67 mg/kg of ligustilide, 3.18 mg/kg of senkyunolide A, and 1.15 mg/kg of ferulic acid for WCR). Blood samples (0.3 mL) were collected in heparinized Eppendorf tubes from suborbital vein before dosing and at 0.033, 0.083, 0.167, 0.25, 0.5, 1, 2, 4, 8, 12, 24, 36, and 48 h after oral administration. The samples were immediately centrifuged at 13,000 rpm for 10 min at room temperature. The plasma was separated and frozen at −20°C until analysis.

### 3.7. Statistical Analysis

The pharmacokinetic parameters including the maximum plasma concentration (*C*_max_), the time to reach maximum concentration (*T*_max_), the area under the concentration-time curve from time zero to the last measured concentration (*AUC*_0*⟶*t_), the area under the concentration-time curve extrapolated to infinity (*AUC*_0⟶∞_), elimination half-life (*T*_1/2_), apparent volume of distribution (*V*_d_), mean residence time (*MRT*), and total body clearance (*CL*) were calculated using WinNonlin software (version 6.0, Pharsight Corporation, CA, USA) using noncompartment model. All values were expressed as the mean ± SD. Comparisons between different groups were performed using the unpaired Student's *t*-test. A value of *P* < 0.05 was considered statistically significant.

## 4. Results and Discussion

### 4.1. Optimization of Chromatographic and Mass Spectrometric Conditions

To achieve sensitive analytical conditions, some chromatographic conditions were optimized, such as types of chromatographic columns, mobile phase compositions, column temperature, and flow rate of the mobile phase. Ultimately, an ACQUITY BEH C_18_ column was selected to separate the analytes and IS to give symmetrical peak responses. In addition, acetonitrile (*A*) and 0.01% (*v/v*) aqueous formic acid (B) were chosen as the mobile phases with gradient elution for a higher response of all analytes. The flow rate was 0.3 mL/min, and the column temperature was optimized to be 40°C.

**Figure 1:**
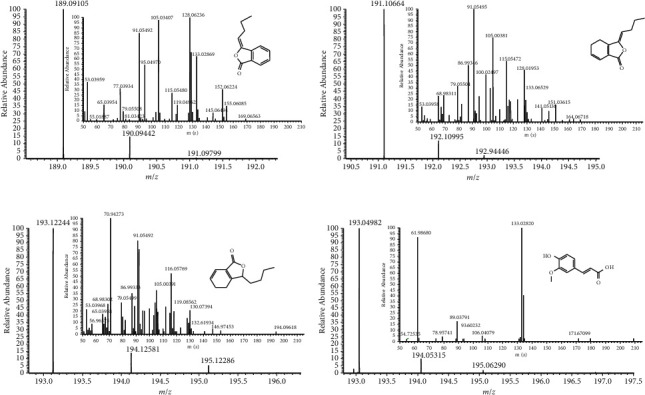
The mass spectra of molecular ions and fragmentation of butylidenephthalide (a), ligustilide (b), senkyunolide A (c), and ferulic acid (d).

After the optimization of mass spectrometry conditions including spray voltage, the flow rate of sheath gas and capillary temperature, butylidenephthalide, ligustilide, senkyunolide A, and IS showed a higher sensitivity in the positive mode ([*M*+*H*]^+^), and on the contrary, ferulic acid showed a higher sensitivity in the negative mode ([M－H]－). Butylidenephthalide, ligustilide, senkyunolide A, ferulic acid, and IS were detected at *m/z* 189.09105, 191.10664, 193.12244, 193.04982, and 447.12817, respectively, within a mass error of 10 ppm. The product mass spectra of the analytes are presented in Figure 1.

### 4.2. Determination of Four Compounds in Oral Liquid Medicine of RCR and WCR

Treated with solid-phase extraction, oral liquid medicines of RCR and WCR were injected into UHPLC-MS/MS system for analysis. The contents of butylidenephthalide, ligustilide, senkyunolide A, and ferulic acid in oral liquid medicine of RCR and WCR were determined at 7.75, 3.01, 116.21, and 13.81 µg/g for RCR and 9.51, 27.89, 132.40, and 48.09 µg/g for WCR, respectively. The results showed that the contents of four analytes in oral liquid medicine of WCR were higher than in RCR. We suspect that there are two main reasons for this phenomenon. First, wine processing may enhance the solubilization of chemical compounds. Second, in a previous study, we found that some phenolic acids like ferulic acid also existed in wine, and a certain amount of ferulic acid could be introduced into WCR through the addition of wine.

### 4.3. The Evaluation of MCAO Rat Model

**Figure 2:**
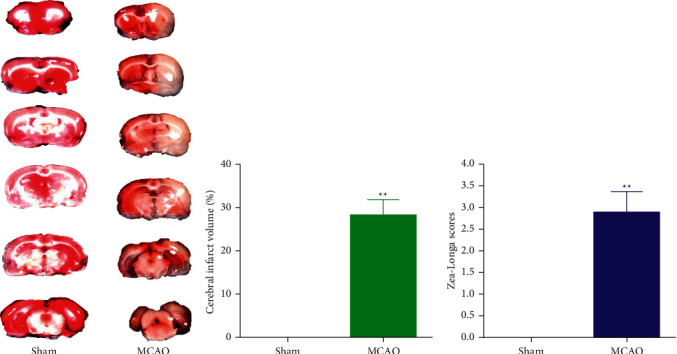
TTC staining results of the representative brain tissue (a), cerebral infarct volume (b), and Zea-Longa scores (c). Compared to sham group, ^∗∗^*P* < 0.01.

The results of Zea-Longa score and TTC staining are shown in Figure 2, which showed that significant cerebral infarction occurred in the model group, and the infarct volume was about 28%. Moreover, MCAO rats exhibited severe neurological deficits with a Zea-Longa score between 2 and 4. The results mentioned above indicated that the model has been successfully established.

## 5. Method Validation

### 5.1. Specificity

**Figure 3:**
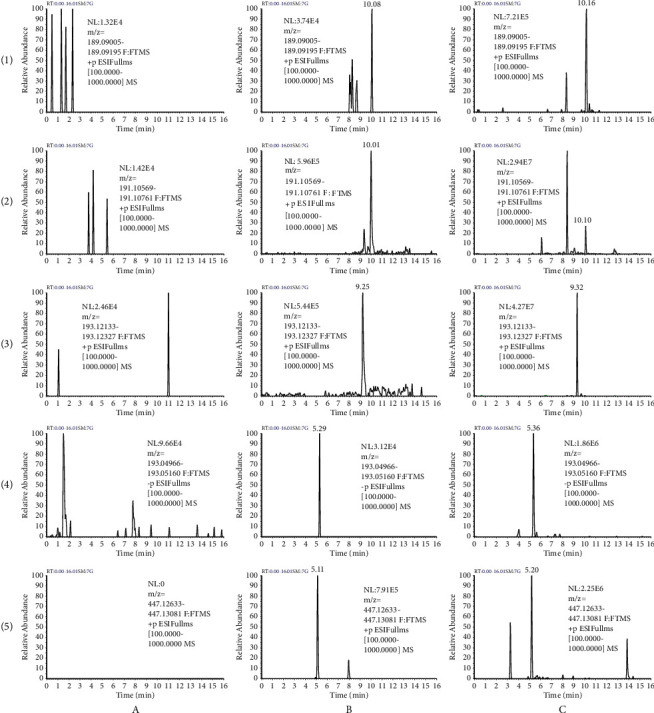
Representative chromatograms of butylidenephthalide (1), ligustilide (2), senkyunolide A (3), ferulic acid (4), and calycosin-7-O-*β*-D-glucoside (IS) (5) in rat plasmas: (a) blank plasma samples; (b) blank plasma samples spiked with butylidenephthalide, ligustilide, senkyunolide A, and ferulic acid at LLOQ and IS; and (c) plasma samples obtained at 1 h after oral administration of RCR at a dose of 24.01 g/kg.

The typical chromatograms of blank plasma from six rats, blank plasma spiked with analytes at LLOQ and IS, and plasma samples at 1 h after oral administration of RCR are shown in Figure 3. The retention times of butylidenephthalide, ligustilide, senkyunolide A, ferulic acid, and IS were approximately 10.15, 10.11, 9.32, 5.36, and 5.18 min, respectively. No significant interferences from endogenous substances and metabolites in blank plasma were observed at the retention times of the analytes and IS.

### 5.2. Linearity and LLOQ

The regression equations, correlation coefficients, and linear ranges for all analytes in plasma are listed in Table 1. All calibration curves were linear over the concentration ranges with correlation coefficients *R*^*2*^ ≥ 0.99. The LLOQ of butylidenephthalide, ligustilide, senkyunolide A, and ferulic acid were 2.38, 3.25, 3.00, and 6.50 ng/mL, respectively. The precision of LLOQ was from 6.24% to 11.08%, and the accuracy of LLOQ was from −3.50% to 7.74%. Details on the assay performance data are listed in Table 2.

### 5.3. Precision and Accuracy

Precision and accuracy of intraday and interday for four analytes in rat plasma are shown in Table 2. The interday and intraday precision results were below 11.51%. The intraday accuracy was from −3.50% to 9.00%, and interday accuracy was from −1.41% to 8.24%, respectively. All the values were within an acceptable range, which indicated that the present method was reliable and reproducible.

### 5.4. Extraction Recovery and Matrix Effect

The extraction recovery and matrix effect are presented in Table 3. The extraction recoveries of butylidenephthalide, ligustilide, senkyunolide A, ferulic acid, and IS were from 94.95% to 110.39%, which suggested that analytes and IS could be effectively extracted from the biosamples. The matrix effect was in the range of 93.84% to 109.73%, which manifested that no significant matrix effect was observed in this method.

### 5.5. Stability

The stability under various storage and process conditions is summarized in Table 4. The results were all within an acceptable limit, illustrating that four analytes in biological samples were stable in the situations described above.

### 5.6. Pharmacokinetic Studies

**Figure 4:**
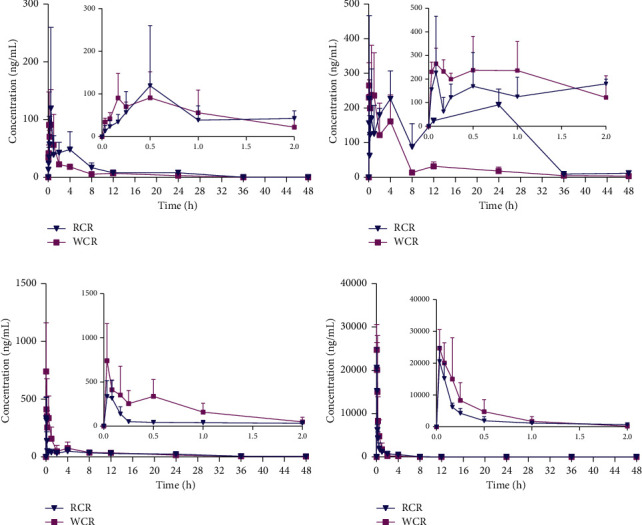
Mean plasma concentration-time curves of butylidenephthalide (a), ligustilide (b), senkyunolide A (c), and ferulic acid (d) after oral administration of RCR and WCR to MCAO rats (mean ± SD, *n* = 6).

The validated UHPLC–MS/MS method was successfully applied to a comparative pharmacokinetic study of butylidenephthalide, ligustilide, senkyunolide A, and ferulic acid after oral administration of RCR and WCR in MCAO rats. The mean plasma concentration-time profiles are illustrated in Figure 4. The major pharmacokinetic parameters are summarized in Table 5.

As seen in Figure 4 and Table 5, for senkyunolide A and ferulic acid, the *C*_max_, *AUC*_0⟶t_, and *AUC*_0⟶∞_ values in WCR were higher than in RCR, which were the completely opposite of our previous pharmacokinetic studies in normal rats [24]. For *C*_max_ of butylidenephthalide and ligustilide, there was no significant difference between WCR and RCR, which was also different from the previous study in normal rats. In normal rats, compared to RCR, the *AUC* (both *AUC*_0⟶t_ and *AUC*_0⟶∞_) and *C*_max_ values of butylidenephthalide, ligustilide, senkyunolide A, and ferulic acid in the WCR group were significantly decreased, even though their contents in oral liquid medicine of WCR were all higher than RCR. At the same time, the *V*_*d*_ and *CL* values were increased in WCR. We guess that, in normal rats, the membrane penetration rate of a molecule was the rate-limiting process, and after processed by rice wine, the promoting blood circulation function of *Chuanxiong Rhizoma* was enhanced, leading to improving the exchange of chemicals between the blood and the tissues and increasing metabolism and excretion. However, in MCAO rats, the rate of blood flow was the rate-limiting process, and when processed by rice wine, the enhanced function of promoting blood circulation could increase the absorption of ingredients. Therefore, the *C*_max_, *AUC*_0⟶t_, and *AUC*_0⟶∞_ values of senkyunolide A and ferulic acid in WCR were all increased.

Besides, the pathological state of cerebral ischemia may change the structure of intestinal flora and the activity of the CYP450 (cytochrome P450) enzyme, thus affecting the metabolism of active ingredients *in vivo* [28], but the specific mechanism remains to be further studied.

To sum up, WCR may have a better efficacy for higher *C*_max_ and *AUC* of some compounds in MCAO rats rather than in normal rats. The MCAO rats could be used to reasonably evaluate the wine-processing effect of *Chuanxiong Rhizoma*.

## 6. Conclusions

An established UHPLC-MS method was successfully applied to the comparative pharmacokinetic study of butylidenephthalide, ligustilide, senkyunolide A, and ferulic acid in MCAO rats after oral administration of RCR and WCR. The results showed the theory of traditional Chinese medicine that the wine processing could enhance the efficiency of Chuanxiong Rhizoma that may be better reflected in model rats rather than in normal rats for the reason that the Cmax and AUC of some compounds in the WCR group were higher than in RCR group only in model rats. This also reminded us whether we want to reasonably evaluate the processing method, and appropriate animal models should be used. This study provided the basis for wine-processing mechanism research of *Chuanxiong Rhizoma* and offered a meaningful guide for WCR when used in the clinical treatment of cerebral ischemia stroke.

## Figures and Tables

**Table 1:** Calibration curves and linearity for butylidenephthalide, ligustilide, senkyunolide A, and ferulic acid in rat plasma.

Analytes	Standard curve	Correlation coefficient (*R*^2^)	Linear range (ng/mL)
Butylidenephthalide	*y* = 0.1818*x* + 0.0025	0.9991	2.38–950.00
Ligustilide	*y* = 1.6324*x* + 0.1124	0.9993	3.25–1300.00
Senkyunolide A	*y* = 2.181*x* + 0.1721	0.9992	3.00–1200.00
Ferulic acid	*y* = 0.394*x* + 0.0295	0.9991	6.50–2600.00
	*y* = 0.4728x − 0.281	0.9998	130.00–26000.00

**Table 2:** Precision and accuracy of butylidenephthalide, ligustilide, senkyunolide A, and ferulic acid in rat plasma (6 replicates per day for 3 days).

Analytes	Nominal concentration (ng/mL)	Intraday		Interday	
		Precision (RSD, %)	Accuracy (RE, %)	Precision (RSD, %)	Accuracy (RE, %)
Butylidenephthalide	2.38	6.24	4.77	6.54	7.02
	4.75	6.30	3.03	7.32	-1.05
	95.00	11.51	9.00	9.11	8.24
	760.00	5.00	8.56	7.04	7.39

Ligustilide	3.25	6.48	1.51	8.52	5.03
	6.50	2.53	-3.14	3.45	-1.41
	130.00	3.58	3.17	7.05	2.62
	1040.00	3.10	5.96	3.59	3.78

Senkyunolide A	3.00	9.68	-3.50	8.50	4.25
	6.00	5.44	3.61	5.13	5.88
	120.00	9.35	2.25	7.71	2.12
	960.00	4.01	2.21	5.37	2.11

Ferulic acid	6.50	11.08	4.69	7.54	7.74
	13.00	5.40	1.99	5.57	-0.96
	1300.00	7.08	3.44	8.25	5.97
	20800.00	4.78	1.73	4.47	2.21

**Table 3:** Extraction recovery and matrix effect of butylidenephthalide, ligustilide, senkyunolide A, ferulic acid, and IS in rat plasma (*n* = 6).

Analytes	Nominal concentration (ng/mL)	Extraction recovery		Matrix effect	
		Mean (%)	RSD (%)	Mean (%)	RSD (%)
Butylidenephthalide	4.75	110.39	8.03	100.52	6.65
	95.00	99.28	6.22	105.68	10.61
	760.00	94.95	3.94	98.09	7.22

Ligustilide	6.50	95.19	5.02	99.24	8.77
	130.00	96.44	8.12	109.73	7.80
	1040.00	95.03	10.89	103.34	1.99

Senkyunolide A	6.00	97.33	4.77	104.34	7.65
	120.00	97.01	5.81	96.64	8.57
	960.00	105.12	9.25	104.02	5.84

Ferulic acid	13.00	96.74	4.26	93.84	9.52
	1300.00	101.71	8.88	99.42	4.77
	20800.00	103.65	6.59	94.13	9.97

IS	140.00	97.73	6.91	97.89	1.43

**Table 4:** Stability of butylidenephthalide, ligustilide, senkyunolide A, and ferulic acid in rat plasma (*n* = 6).

Analytes	Added (ng/mL)	Short-term stability		Long-term stability		Postpreparative stability		Freeze-thaw stability	
		RSD (%)	RE (%)	RSD (%)	RE (%)	RSD (%)	RE (%)	RSD (%)	RE (%)
Butylidenephthalide	4.75	7.83	-3.45	7.95	6.25	6.33	-3.90	7.62	-3.42
	95.00	7.39	8.30	9.38	8.60	9.37	7.57	7.30	1.77
	760.00	5.15	4.26	9.31	4.94	9.54	5.09	8.81	7.12

Ligustilide	6.50	9.22	1.93	5.43	3.40	3.47	1.06	5.26	2.58
	130.00	5.19	9.54	4.78	1.21	6.60	2.08	3.81	2.70
	1040.00	10.20	0.46	6.12	1.97	4.52	3.09	8.87	10.56

Senkyunolide A	6.00	6.69	6.95	4.28	8.99	4.18	5.84	5.60	6.96
	120.00	5.86	1.98	6.16	3.78	7.05	2.08	4.67	9.38
	960.00	4.03	4.71	5.54	5.83	5.61	3.04	5.91	1.97

Ferulic acid	13.00	4.59	3.38	4.02	3.26	3.49	-5.34	5.27	1.76
	1300.00	9.36	-7.50	7.52	-2.85	9.10	7.93	7.08	-4.12
	20800.00	5.18	2.44	7.13	-4.05	2.90	1.44	2.83	2.01

**Table 5:** Pharmacokinetic parameters of butylidenephthalide, ligustilide, senkyunolide A, and ferulic acid after oral administration of RCR and WCR at a dose of 24.01 g/kg to MCAO rats (mean ± SD, *n* = 6).

Parameter	Butylidenephthalide		Ligustilide		Senkyunolide A		Ferulic acid	
	RCR	WCR	RCR	WCR	RCR	WCR	RCR	WCR
*AUC*_0⟶t_ (h^*∗*^ng/mL)	472.65 ± 143.27	298.45 ± 106.82	5508.21 ± 688.91	1621.17 ± 435.60^*∗∗*^	1045.86 ± 175.86	1380.79 ± 277.53	8387.99 ± 2105.89	9621.64 ± 3523.69
*AUC*_0⟶∞_ (h^*∗*^ng/mL)	571.48 ± 151.62	319.16 ± 112.26^*∗*^	5750.77 ± 872.10	1689.50 ± 436.08^*∗∗*^	1092.92 ± 175.24	1447.90 ± 289.33^*∗*^	8491.91 ± 2088.56	9684.96 ± 3520.26
*CL* (mL/h)	92.21 ± 26.95	170.23 ± 42.02^*∗∗*^	3.45 ± 0.56	92.46 ± 23.32^*∗∗*^	694.70 ± 121.20	511.11 ± 131.92	10.93 ± 2.90	30.59 ± 13.96^*∗*^
*C*_max_ (ng/mL)	130.68 ± 134.43	125.03 ± 51.25	338.31 ± 173.42	319.29 ± 63.66	417.83 ± 217.82	875.17 ± 335.22^*∗*^	22056.42 ± 2146.68	27503.81 ± 4757.40^*∗*^
*T*_1/2_ (h)	8.75 ± 4.54	9.07 ± 2.49	9.18 ± 2.30	12.36 ± 4.84	9.07 ± 1.26	12.02 ± 2.27^*∗*^	7.28±2.22	8.54 ± 4.01
*MRT* (h)	12.54 ± 5.23	9.70 ± 2.39	18.10 ± 0.43	11.36 ± 0.94^*∗∗*^	16.39 ± 1.55	12.65 ± 1.26^*∗∗*^	4.47 ± 0.86	2.49 ± 0.79^*∗∗*^
*T*_max_ (h)	0.32 ± 0.18	0.33 ± 0.16	10.52 ± 12.40	0.61 ± 0.41	0.05 ± 0.03	0.07 ± 0.06	0.04 ± 0.02	0.07 ± 0.06
*V*_d_ (mL)	1167.87 ± 682.81	2175.01 ± 611.90^*∗*^	46.22 ± 15.43	1642.57 ± 700.21^*∗∗*^	9061.04 ± 1764.31	8789.77 ± 2562.16	114.16 ± 37.23	388.53 ± 281.92

Compared to RCR group, ^*∗*^*P* < 0.05 and ^*∗∗*^*P* < 0.01.

## Data Availability

The data used to support the findings of this study are included within the article.

## Abbreviations

*AUC*_0*⟶*t_: The area under the concentration-time curve from time zero to the last measured concentration
*AUC*_0⟶∞_: The area under the concentration-time curve extrapolated to infinity
CCA: Common carotid artery
*CL*: Total body clearance
*C*_max_: Maximum plasma concentration
CYP450: Cytochrome P450
ECA: External carotid artery
ICA: Internal carotid artery
IS: Internal standard
LLOQ: Linearity and lower limit of quantification
MCA: Middle cerebral artery
MCAO: Middle cerebral artery occlusion
*MRT*: Mean residence time
QC: Quality control
RCR: Raw *Chuanxiong Rhizoma*
RE: Relative error
RSD: Relative standard deviation
*T*_1/2_: Elimination half-life
TCM: Traditional Chinese medicine
*T*_max_: The time to reach maximum concentration
TTC: 2,3,5-Triphenyltetrazolium chloride
UHPLC-MS/MS: Ultrahigh performance liquid chromatography tandem mass spectrometry
*V*_d_: Apparent volume of distribution
WCR: Wine-processed *Chuanxiong Rhizoma.*
